# Transient patterns of advanced brain ageing in female adolescents with anorexia nervosa

**DOI:** 10.1192/bjp.2024.119

**Published:** 2024-11

**Authors:** Tatiana Stratton, Klaas Bahnsen, Daniel Geisler, Fabio Bernardoni, Christian Gaser, Stefan Ehrlich, Esther Walton

**Affiliations:** Department of Psychology, University of Bath, Bath, UK; Translational Developmental Neuroscience Section, Division of Psychological and Social Medicine and Developmental Neurosciences, Faculty of Medicine, Technische Universität Dresden, Dresden, Germany; Structural Brain Mapping Group, Department of Neurology, Jena University Hospital, Jena, Germany; Department of Psychiatry and Psychotherapy, Jena University Hospital, Jena, Germany; German Center for Mental Health (DZPG), Jena-Magdeburg-Halle, Germany; Translational Developmental Neuroscience Section, Division of Psychological and Social Medicine and Developmental Neurosciences, Faculty of Medicine, Technische Universität Dresden, Dresden, Germany; Eating Disorders Research and Treatment Center at the Dept. of Child and Adolescent Psychiatry, Faculty of Medicine, Technische Universität Dresden, Dresden, Germany

**Keywords:** Brain age, neuroimaging, anorexia nervosa, longitudinal, recovery

## Abstract

**Background:**

Anorexia nervosa is a psychiatric disorder characterised by undernutrition, significantly low body weight and large, although possibly transient, reductions in brain structure. Advanced brain ageing tracks accelerated age-related changes in brain morphology that have been linked to psychopathology and adverse clinical outcomes.

**Aim:**

The aim of the current case–control study was to characterise cross-sectional and longitudinal patterns of advanced brain age in acute anorexia nervosa and during the recovery process.

**Method:**

Measures of grey- and white-matter-based brain age were obtained from T1-weighted magnetic resonance imaging scans of 129 acutely underweight female anorexia nervosa patients (of which 95 were assessed both at baseline and after approximately 3 months of nutritional therapy), 39 recovered patients and 167 healthy female controls, aged 12–23 years. The difference between chronological age and grey- or white-matter-based brain age was calculated to indicate brain-predicted age difference (BrainAGE_GM_ and BrainAGE_WM_).

**Results:**

Acute anorexia nervosa patients at baseline, but not recovered patients, showed a higher BrainAGE_GM_ of 1.79 years (95% CI [1.45, 2.13]) compared to healthy controls. However, the difference was largely reduced for BrainAGE_WM_. After partial weight restoration, BrainAGE_GM_ decreased substantially (beta = −1.69; CI [−1.93, −1.46]). BrainAGEs were unrelated to symptom severity or depression, but larger weight gain predicted larger normalisation of BrainAGE_GM_ in the longitudinal patient sample (beta = −0.65; CI [−0.75, −0.54]).

**Conclusions:**

Our findings suggest that in patients with anorexia nervosa, undernutrition is an important predictor of advanced grey-matter-based brain age, which itself might be transient in nature and largely undetectable after weight recovery.

Anorexia nervosa is a severe psychiatric disorder characterised by significantly low body weight, intense fear of weight gain and cognitive disturbances regarding body shape. In the last two decades, advances in neuroimaging, including the analysis of T1-weighted magnetic resonance imaging (MRI), have yielded some valuable insights into the neurological alterations associated with anorexia nervosa.^1^ One of the most well-established findings is a global reduction in grey matter volumes, which is particularly significant among adolescents in comparison to adults.^1–4^ Reductions in white matter volumes and integrity have also been reported, albeit to a lesser extent to those observed in grey matter and findings are considerably less robust across ages.^1,5,6^

The developmentally patterned and potentially transient character of these alterations in anorexia nervosa over the life course is intriguing. Our brains undergo numerous structural changes during development and subsequent ageing. These neural changes are characterised by synaptic reorganisation and elimination, dendritic pruning, de-/myelination and cell body shrinkage.^7,8^ Specifically, grey matter volumes show a steady decrease from childhood onwards, while white matter volume follows an inverted ‘U-shape’, peaking in early adulthood.^8,9^ This could explain why grey matter alterations in anorexia nervosa are more easily detectable than white matter reductions in adolescence and across development.

Brain ageing is a normal neurological process that accompanies typical grey matter and white matter brain maturation and ageing. Advanced brain age – an ‘older’ predicted brain age compared to chronological age – has been linked to negative long-term neurologic and somatic outcomes.^7^ There is emerging evidence that advanced brain age might also be a promising trait marker for various psychiatric disorders in adulthood.^10–14^ The simplicity of being a singular measure, which is nevertheless able to capture the cumulative and often correlated morphological changes in multiple brain regions across age, and its strong links with future clinical outcomes,^15^ makes brain age a potentially better marker for psychopathology compared to studying numerous, distinct morphological features individually (e.g. region-specific cortical thickness, surface area or volume).

However, despite its simplicity and clinical utility, two key questions remain. First, it is not yet clear to what extent anorexia-nervosa-specific trends in advanced brain ageing can be captured in adolescence – a time period in which the brain is still undergoing important developmental changes. While some studies have characterised brain ageing in adolescence,^16–21^ it is still unknown whether delayed or advanced brain age in this developmental period links to anorexia nervosa.

Second, it is unclear whether advanced brain ageing in anorexia nervosa might be driven by co-occurring, yet more transient influences. For example, significant controversy exists around the reversibility of structural brain changes in anorexia nervosa with recovery and weight gain.^5^ Some studies have indicated a reversibility of grey matter structural alterations in anorexia nervosa with weight restoration, suggesting that grey matter reductions could be a consequence of starvation.^1^ Conversely, there is little evidence for the reversibility of white matter, thus suggesting that white matter alterations may be more structurally ingrained and possible trait markers for disorder prognosis,^2^ relating less to the course of weight development. Several factors such as body mass index (BMI) at admission, illness severity and illness duration likely determine the extent of this reversibility, but the degree to which this applies to a singular measure such as advanced brain ageing is still unclear. The goal of the current study was to investigate whether grey matter and white matter brain age is altered (delayed or advanced) in predominately adolescent anorexia nervosa patients, and if this is a transient process that fluctuates with nutritional rehabilitation. The findings will contribute to a better understanding of the stability of brain ageing in anorexia nervosa and the role of severe undernutrition in this process.

## Method

### Participants

The total sample consisted of 431 scans of 335 female volunteers: 129 acutely underweight anorexia nervosa patients (acAN_baseline_) diagnosed according to DSM-IV^22^ criteria (age range = 12.1–22.1 years), 39 recovered anorexia nervosa patients (recAN; age range = 15.6–22.9 years) and 167 healthy control participants (healthy control; age range = 12.1–22.9 years; see Table 1 and Supplementary Material Fig. 1 available at https://doi.org/10.1192/bjp.2024.119). We carefully compiled the healthy control sample according to age in an attempt to obtain an independent age-matched case–control sample for each patient group (acAN_baseline_, acAN_follow-up_, recAN) (see Supplementary Material Section 1.1). We assert that all procedures contributing to this work comply with the ethical standards of the relevant national and institutional committees on human experimentation and with the Helsinki Declaration of 1975, as revised in 2008. All procedures involving human participants/patients were approved by the institutional review board of the Technische Universität Dresden (approval numbers EK 14012011 and EK 536122015) and all participants (or their legal guardians, if underage) gave written informed consent.

**Table 1:** Brain age, demographic and clinical measures

	Mean (s.d.)						
	Healthy control	acAN_baseline_	acAN_follow-up_	recAN	*p*_cross-sectional_	*Post hoc* tests	*p*_longitudinal_
*N*	167	129	95	39			
Age	17.01 (2.88)	15.77 (2.20)	15.87 (2.06)	19.77 (2.07)	<0.001	Healthy control > acAN_baseline_ Healthy control < recAN acAN_baseline_ < recAN	<0.001
BMI-SDS	−0.05 (0.71)	−3.07 (1.18)	−0.70 (0.60)	−0.52 (0.50)	<0.001	Healthy control > acAN_baseline_ Healthy control > recAN acAN_baseline_ < recAN	<0.001
Duration of illness	Not applicable	1.90 (1.52)	1.86 (1.06)	Not available	Not applicable	Not applicable	<0.001
BDI-II total score	4.81 (5.19)	21.24 (10.97)	12.50 (9.87)	9.44 (7.69)	<0.001	Healthy control < acAN_baseline_ Healthy control < recAN acAN_baseline_ > recAN	<0.001
EDI-2 total score	141.05 (29.40)	204.96 (44.48)	183.69 (43.73)	173.28 (36.49)	<0.001	Healthy control < acAN_baseline_ Healthy control < recAN acAN_baseline_ > recAN	<0.001
BrainAGE_GM_	−0.05 (1.36)	1.74 (1.65)	0.16 (1.28)	−0.48 (1.28)	<0.001	Healthy control < acAN_baseline_ acAN_baseline_ > recAN	<0.001
BrainAGE_WM_	−0.01 (1.19)	−0.40 (1.22)	−0.34 (1.15)	−0.46 (1.08)	0.008	Healthy control > acAN_baseline_	0.018

acAN_baseline_, acute anorexia nervosa patients at baseline; acAN_follow-up_, acute anorexia nervosa patients after partial recovery; recAN recovered patients; BMI-SDS, body mass index standard deviation score; BDI-II, Beck Depression Inventory-II; EDI-2, Eating Disorder Inventory-2; BrainAGE_GM_, grey-matter brain-predicted age difference; BrainAGE_WM_, white-matter brain-predicted age difference.

Cross-sectional *P*-values are based on linear regression models; longitudinal *P*-values are based on linear mixed models accounting for group as a fixed effect and participant as a random effect.

The acAN patients (duration of illness = 1.9 ± 1.5 years, range = 0.2–9.5 years) were admitted to eating disorder programmes at the Technische Universität Dresden. Diagnosis was established according to the Structured Interview for Anorexia and Bulimia Nervosa (SIAB-EX), which requires a BMI < 10th age percentile (if younger than 15.5 years of age) and <17.5 kg/m^2^ (if older than 15.5 years). A total of 95 acAN patients (age range = 12.4–22.2 years) completed all assessments at two time points: first within 96 h after beginning nutritional rehabilitation (acAN_baseline_) and a second time after a BMI increase (and maintenance) of at least 10% (acAN_follow-up_).

To be considered ‘recovered’, recAN participants had to (a) maintain a BMI > 18.5 kg/m^2^ (if older than 18 years of age) or >10th age percentile (if younger than 18 years of age), (b) menstruate and (c) have not binged, purged or engaged in restrictive eating patterns, all for at least 6 months before the study. Healthy control participants were recruited through advertisement among middle school, high school and university students, and eating disorders were excluded using the SIAB-EX. We applied several additional exclusion criteria (see Supplementary Material Sections 1.1 and 1.2) for all groups – most importantly, a history of bulimia nervosa or ‘regular’ binge eating, psychotropic medications other than selective serotonin reuptake inhibitors (SSRIs) within 2 weeks before the study, substance misuse and neurologic or medical conditions.

### Clinical measures

BMI was calculated by dividing body weight (kilograms) by height squared (metres). This study utilised the age-adjusted body mass index standard deviation score (BMI-SDS) for females, calculated according to Cole^23^ and German population reference data.^24,25^ We assessed eating disorder-specific psychopathology with the Eating Disorder Inventory–2 (EDI-2 total score) and affective symptoms with the Beck Depression Inventory–II (BDI-II total score) both at baseline and at follow-up (see Supplementary Material Section 1.2). Demographic and clinical data were managed using a web-based tool (Research Electronic Data Capture [REDCap], version 14.0.41; Vanderbilt University, Nashville, USA; see http://www.project-redcap.org ).

### Magnetic resonance imaging acquisition and processing

All participants underwent MRI scanning between 8.00 and 9.00 h following an overnight fast. High-resolution three-dimensional T1-weighted structural scans were acquired on a 3.0 T scanner (MAGNETOM Trio, Siemens, Erlangen, Germany) using a magnetisation prepared rapid acquisition gradient-echo sequence (Supplementary Material Section 1.3).

For quality control, a tessellated reconstruction of the cerebral cortex was obtained in an automated manner using the FreeSurfer software suite, version 5.3.0 (Laboratory for Computational Neuroimaging at the Athinoula A. Martinos Center for Biomedical Imaging, Boston, USA; see http://surfer.nmr.mgh.harvard.edu).

Next, all images were subjected to further processing with the FreeSurfer longitudinal stream. Participants with scan artefacts significantly affecting parcellation were excluded from the analysis (see Supplementary Material Section 1.3).

### BrainAGE score analysis and age estimation framework

The BrainAGE framework used a machine-learning pattern recognition method called relevance vector regression (RVR). RVR was recently used to model healthy brain ageing and subsequently estimate individual brain age based on T1-weighed images.^26^ A linear kernel was chosen, as age estimation accuracy has been shown to not improve when using non-linear kernels.^26^

Generally, the age regression model is trained with chronological age and pre-processed whole brain MRI data of the training sample, which results in a complex model of healthy brain ageing. More detailed information and an illustration of the most important features that were used by RVR to model brain ageing can be found in Franke et al.^26^ We applied VBM (version 8, SPM8, Jena, Germany; see https://neuro-jena.github.io/software.html#vbm) to segment MRI data into local maps of grey and white matter and smoothed the affine transformed grey and white matter segmentations with a Gaussian kernel of 8 mm. For training our BrainAGE framework we used 226 female participants within an age range of 12–23 years from the National Institutes of Health (NIH) MRI study of normal brain development (release 4, *n* = 876, males/females = 411/465, age range 5–23 years^27^) and resampled training and test data to a spatial resolution of 8 mm after smoothing with a Gaussian kernel of 8 mm. The brain age of a test subject can be subsequently estimated using their structural MRI data to combine the complex, multidimensional ageing pattern across the whole brain into a singular value. The brain age is then calculated by applying the regression pattern of healthy brain ageing and combining all voxel-wise information across the whole brain.^10^ The difference between estimated brain age and chronological age produces the BrainAGE score (in the current study referred to as ‘BrainAGE_GM_’ (grey matter) and ‘BrainAGE_WM_’ (white matter)). A positive value indicates advanced brain ageing and an ‘older’ brain than expected, while a negative value indicates decelerated brain ageing and a ‘younger’ brain.^19^ The greater the value (above or below zero), the greater the deviation of brain age from chronological age. As it is known that the BrainAGE prediction can be subject to an age bias, resulting in overestimation of younger participants and underestimation of older participants, we need to correct for this age bias.^28^ Age-bias correction ensures that any group comparisons or associations with other parameters of interest are not influenced by the age-dependence of the predictions. Therefore, all brain age values were finally adjusted for age effects by correcting for the linear trend in BrainAGE using only control data and applying this trend-correction to all data.

### Statistical analysis

First, we investigated cross-sectional group differences (acAN_baseline_, recAN, healthy control) in both grey and white matter BrainAGE, measured in years, using a linear regression (cross-sectional model 1). In secondary models, we included age and age × diagnosis as a covariate to account for potential variation in BrainAGE because of these factors (cross-sectional model 2a). Specifically, the age term covaried for any main effect of age (e.g. advanced brain ageing might only become apparent in older individuals). The age × diagnosis interaction effect was included to account for differential age differences among the groups. We also explored the possible effects of current or recent SSRI use (cross-sectional model 2b) and anorexia nervosa subtype (restricting versus binge/purge; cross-sectional model 2c). Finally, we assessed the degree to which clinical measures, including BMI, eating disorder symptom severity, depression scores and duration of illness, were associated with grey and white matter BrainAGE (cross-sectional model 3). To account for multiple testing (two BrainAGE measures and three models), we defined statistical significance at *p* = 0.05/6 = 0.0083.

In the longitudinal analyses, we applied linear mixed models to assess the degree of change in BrainAGE over time, accounting for group as a fixed effect and participant as a random effect (longitudinal model 1) and additionally for clinical measures at baseline (longitudinal model 2). Finally, we assessed the degree to which change in clinical measures over time predicts change in BrainAGE (longitudinal model 3). Statistical significance for these longitudinal analyses was corrected for two BrainAGE measures and three models (*P* < 0.0083). All analyses were carried out using R (version 3.6.1; see https://www.r-project.org/).

## Results

### Sample characteristics

As expected, healthy control, acAN and recAN participants differed in BMI-SDS, eating disorder symptoms and depression scores; and symptom severity reduced in acAN participants from baseline to follow-up (Table 1). In all three measures, acAN_baseline_ participants showed the largest deviations from healthy control participants with smaller, yet still significant differences between acAN_follow-up_ or recAN and healthy control participants. The acAN participants were slightly younger than control participants at both time points (1.24 years at baseline and 1.14 years at follow-up), while recAN participants were 2.76 years older than control participants (Supplementary Material Fig. 2).

### BrainAGE associations with chronological age

Overall, the structural brain age measures predicted chronological age with a mean absolute error of 1.33 years (s.d. = 1.10) for grey matter derived brain age, and 0.97 years (s.d. = 0.74) for white matter derived brain age. Both brain age measures strongly correlated with chronological age (*r*_GM_ = 0.81; *r*_WM_ = 0.91) and with each other (*r* = 0.84). However, the difference in chronological age between both measures (i.e. BrainAGE_GM_ and BrainAGE_WM_) was less strongly correlated (*r* = 0.44; Supplementary Material Fig. 3), suggesting that grey- and white-matter-based measures captured unique aspects of brain ageing. See Table 1 and Supplementary Material Fig. 2 for BrainAGE and clinical descriptives for each group.

### Cross-sectional group differences in BrainAGE

**Fig. 1:**
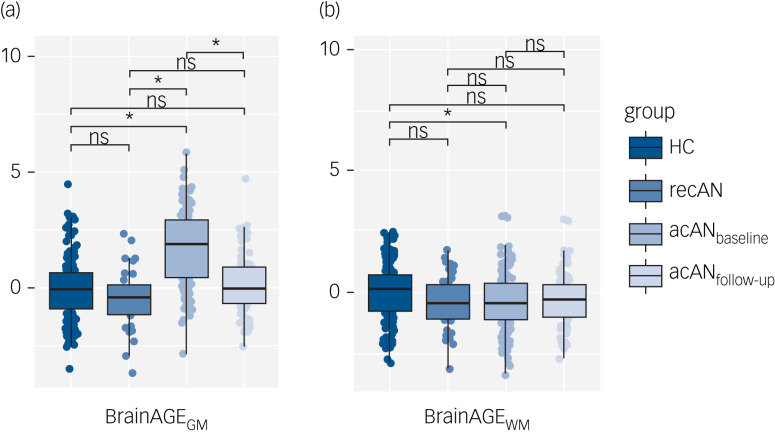
(a) Grey-matter- and (b) white-matter-based predicted brain age differences across groups. acAN_baseline_, acute anorexia nervosa patients at admission; acAN_follow-up_, acute anorexia nervosa patients after partial weight recovery; recAN, recovered patients; BrainAGE_GM_, grey-matter brain-predicted age difference; BrainAGE_WM_, white-matter brain-predicted age difference; ns, not significant. *Significant at *P* < 0.0083.

We observed strong differences in BrainAGE_GM_, but much weaker effects in BrainAGE_WM_ among acAN_baseline_, recAN and healthy control participants (Fig. 1, Supplementary Material Fig. 2 and Supplementary Material Table 1). Pair-wise contrasts indicated acAN_baseline_ patients had a significantly ‘older’ BrainAGE_GM_ (beta = 1.79 years; 95% CI [1.45–2.13]; *p* < 0.001) compared to control participants. However, differences in BrainAGE_GM_ between recovered patients and healthy control participants were largely alleviated (beta = −0.43 years; CI [−0.94 to −0.09]; *p* = 0.103). These effects remained stable when also covarying for age and age by group interaction (Supplementary Material Table 2), as well as SSRI use (Supplementary Material Table 3) and anorexia nervosa subtype (Supplementary Material Table 4).

The acAN_baseline_ patients had a ‘younger’ BrainAGE_WM_ of 0.39 years (95% CI [−0.67 to −0.12]; *p* = 0.005, Supplementary Material Table 5) compared to control participants, but this difference was largely accounted for by the additional effect of age (Supplementary Material Tables 6–8). There were no meaningful differences in BrainAGE_WM_ between recovered patients and control participants after multiple testing correction (Supplementary Material Tables 5–8).

### Cross-sectional BrainAGE associations with clinical measures

BrainAGE_GM_, but not BrainAGE_WM_, was weakly associated with BMI-SDS in acAN_baseline_ participants (BrainAGE_GM_ beta = −0.34; 95% CI [−0.63 to −0.06]; *p* = 0.017) after accounting for age, eating disorder symptom severity, depression scores and duration of illness. Neither BrainAGE measure was associated with these clinical measures (Supplementary Material Tables 9 and 10).

### Longitudinal change in BrainAGE

**Fig. 2:**
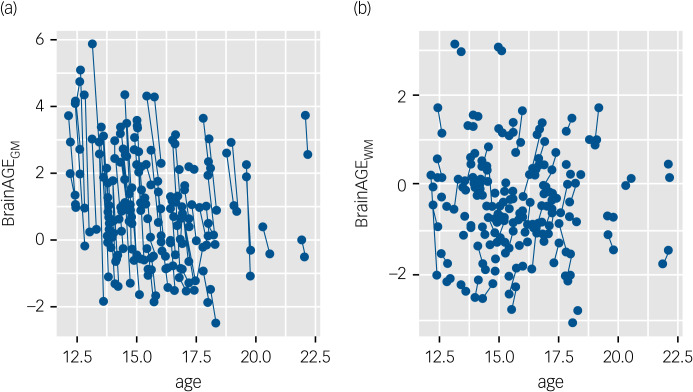
Normalisation of (a) BrainAGE_GM_ and (b) BrainAGE_WM_ during partial weight recovery. BrainAGE_GM_, grey-matter brain-predicted age difference; BrainAGE_WM_, white-matter brain-predicted age difference.

Ninety-five patients were followed up until partial weight recovery (~3 months). Over this period, we observed a large decrease in BrainAGE_GM_ (beta = −1.69; 95% CI [−1.93 to −1.46]; *p* < 0.001; Supplementary Material Table 11), down to the levels observed in control participants (beta = −0.21; 95% CI [−0.57 to 0.15]; *p* = 0.252; Figs. 1(a) and 2(a)). On average, BrainAGE_GM_ decreased by 1.69 years over this time period. This finding remained stable when covarying for clinical measures at baseline (Supplementary Material Table 12). The effect in BrainAGE_WM_ was much weaker and did not survive correction for multiple comparison (Figs. 1(b) and 2(b); Supplementary Material Tables 13 and 14).

### Predictors of BrainAGE change

Weight gain during partial recovery was the only clinical measure that independently predicted BrainAGE_GM_ change (beta = −0.65; 95% CI [−0.75 to −0.54]; *p* < 0.001; Supplementary Material Table 15) over and above duration of illness at baseline and changes in eating disorder symptomatology or depression during recovery. That is, larger weight gain predicted larger normalisation of BrainAGE_GM_. This effect was not observed for white-matter-based brain age (Supplementary Material Table 16).

## Discussion

The current study is, to our knowledge, the first to characterise transient patterns of brain ageing in adolescents with anorexia nervosa and investigate the role of undernutrition in this process. On average, anorexia nervosa patients at baseline showed a significant discrepancy between actual age and predicted age, with a BrainAGE_GM_ of +1.7years. This was significantly higher than the BrainAGE_GM_ observed in healthy control participants, thus indicating advanced brain ageing in anorexia nervosa. No differences in BrainAGE_WM_ were observed between groups. Strikingly, longitudinal follow-up of partially weight recovered acAN patients (after approximately 3 months) revealed a significant reduction in BrainAGE_GM_, returning to levels observed in healthy individuals. In addition, larger weight gain predicted larger normalisation of BrainAGE_GM_.

Our results indicate that age-related structural alterations in grey matter, but not white matter, may resemble a transient advanced brain ageing process in anorexia nervosa. Grey matter reductions, possibly reflective of neuronal remodelling,^29^ are commonly observed in anorexia nervosa patients, specifically in regions within the default mode network and sensorimotor network. In line with our results on BrainAGE_GM_, these previously reported grey matter reductions seem to be largely diminished after weight restoration.^3,29–31^ White matter alterations, a potential measure of glial remodelling, are also observed in anorexia nervosa, albeit to a lesser extent and with weaker signs of reversibility with weight recovery.^32–34^ Hence, the brain ageing effects reported in this study are in line with the patterns of structural grey and white matter alterations previously observed in anorexia nervosa. Furthermore, our findings indicate that normalisation of nutrition and weight status can reverse changes in BrainAGE_GM_ in patients with anorexia nervosa. This is contrary to reports suggesting that anorexia nervosa might be characterised by degenerative neural processes indicative of advanced, more permanent brain ageing.^35,36^

We observed overall advanced – not delayed – brain ageing in our sample of predominately adolescent patients. This is relevant as adolescence is a period in which the brain is still undergoing important developmental changes. As such, it is not clear whether advanced or delayed brain maturation in this developmental period is more likely to be disadvantageous. Previous studies have reported *delayed* brain maturation in children who were born preterm.^19^ In adolescence and early adulthood, *advanced* brain maturation has been linked to better cognition^18,20^ and being female,^37^ but also to schizophrenia,^21^ thus suggesting a complex relationship between brain maturation and health characteristics throughout development.

Our cross-sectional findings compare well to studies investigating brain ageing in other psychiatric disorders. The worldwide largest study on brain age in major depressive disorder (MDD)^13^ reported similar, slightly advanced BrainAGE scores of +1.08 years in patients. Other work reported higher scores than those found in this study, for MDD (+4.0 years), schizophrenia (+3.4 years to +5.5 years) and bipolar disorder (+3.1 years).^11,12,14^ While overall consistent in direction, the variability in effect sizes highlights the meaningful diversity that exists in BrainAGE across and within psychiatric disorders.

Several biological mechanisms may explain the advanced brain ageing observed in this study. For example, inflammation and mitochondrial dysfunction, which are characteristic of natural ageing, might become dysfunctional in anorexia nervosa, especially during the acute stage of disease. In support, a recent study observed a dysregulation of several inflammatory markers in acAN patients, which was also linked to BMI, fat mass and age.^38^ However, other studies were unable to show effects of commonly investigated cytokine concentrations (e.g. IL-6, TNF-α) on vertex-wise cortical thickness, neither cross-sectionally nor longitudinally.^39^ Still, given the large number of pro- and anti-inflammatory molecules that may play a role and additional potential effects of, for example, neurotrophins (which have been shown to be lower in anorexia nervosa^40^), it is entirely possible that altered BrainAGE in anorexia nervosa is mediated by such factors. Similarly, mitochondrial dysfunction and oxidative stress has been observed in young adult anorexia nervosa patients.^41^ It is noteworthy that oxidative stress, which may be caused by dysfunctional mitochondria, can also activate numerous pro-inflammatory markers such as Interleukin-6, which itself is linked to age-related processes.

The findings of the current study should be considered in light of potential limitations. First, the sample consisted solely of young anorexia nervosa patients. Thus, we cannot confidently apply these results to older cohorts with different neurodevelopmental trajectories or other mental health conditions. Moreover, the sample consisted entirely of females, and therefore we are unable to acknowledge any potential gender differences in brain ageing patterns. Also, groups were not matched for chronological age (e.g. the control group was on average younger than the recovered patient group, yet slightly older than the two acute patient groups). Although we covaried for age and age × diagnosis effects to account for these differences, we cannot completely rule out that our findings were partially influenced by age differences among groups. Second, we focussed on the role of weight restoration, but other factors such as hydration, re-feeding and menstrual cycles might be equally important and need to be considered in future studies. Finally, BrainAGE – the difference between an individual's actual and predicted age – is essentially a measure of error. This (mean absolute) error (1.33 and 0.97 years for grey and white matter, respectively) was similar in size or even larger than the group differences that we observed (1.79 and 0.39 years for grey and white matter, respectively). Several factors, such as smoking and technical artefacts, may influence this prediction error and confound results. Furthermore, the interpretation of BrainAGE is not straightforward. It is not a direct measure of chronological age, but rather an assessment of deviation from typical ageing trajectories observed in a healthy population. The observed ‘rejuvenation’ of brain age does not imply a reversal of chronological ageing. Instead, the observed decrease in BrainAGE score reflects a partial reversal of accelerated brain ageing – brain structures may be returning to a state more typical of a person of that chronological age, indicating a reversal of some (pseudo-)atrophy. Thus, the BrainAGE framework provides valuable insight beyond mere loss of brain matter. It quantitatively assesses recovery in brain health and structure, providing a useful biomarker for evaluating the effects of treatment and recovery in disorders such as anorexia nervosa. Nevertheless, effects should be considered with caution. To allow for a more reliable and clinically relevant assessment of BrainAGE in anorexia nervosa, but potentially also across disorders, there is a need to conduct future research using consistent methodologies and larger samples.

In conclusion, compared to healthy control participants, female anorexia nervosa patients show advanced grey matter brain ageing of +1.7 years. This advanced brain ageing in grey matter was transient and largely diminished after weight restoration. The significant deviation from normal brain ageing observed in this study may contribute to and provide some explanation for the negative outcomes associated with anorexia nervosa, including increased risk of mortality. Furthermore, this deviation could potentially be used to identify patients who are at a higher risk of negative long-term psychiatric, neurologic and somatic outcomes, therefore allowing clinicians to provide additional support and intervene as early as possible.^13^ As well as furthering our understanding of the neurobiological underpinnings of anorexia nervosa, this study has provided a significant contribution to our understanding of brain ageing in anorexia nervosa and given us the first insight into the transient patterns of this process. Identifying factors, such as weight gain, that contribute to these patterns will have important implications for developing better treatments for anorexia nervosa.

## Supporting information

Stratton et al. supplementary material Stratton et al. supplementary material

## Data Availability

The data used in this study cannot be publicly deposited. The data that support the findings of this study are available from author S.E., upon reasonable request.
