# Supplementary material for: Transient patterns of advanced brain ageing in female adolescents with anorexia nervosa
Source: Br J Psychiatry. 2024 Nov;225(5):499–505. doi: 10.1192/bjp.2024.119 (PMC11663477; doi:10.1192/bjp.2024.119)
Supplement: Stratton et al. supplementary material [file S0007125024001193sup001.docx]

**Transient patterns of brain ageing in female adolescents with Anorexia Nervosa**

***Supplemental Information***

**Supplemental Methods**

***SM Section 1.1 Participants***

Acute AN participants were recruited from specialized eating disorder programs at the Technische Universität Dresden. Since nutritional rehabilitation has to be carried out with caution at the beginning of the treatment due to possible complications such as refeeding syndrome, our patients had at that stage of treatment a calorie intake of about 1000 to 1500 kcal per day.

A subsample of the original acute AN sample completed all assessments at two time points (longitudinal sample; acAN_follow-up_; SM Figure 1).

Fully recovered patients (recAN) had to meet the following inclusion criteria: 1) maintain a BMI>18.5 kg/m2 (if older than 18 years of age) or >10th age percentile (if younger than 18 years of age); 2) have normal menstruation; and 3) have not binged, purged, or engaged in restrictive eating patterns for at least 6 months before the study.

Healthy individuals (HC) were recruited via advertisements aimed at middle, and high school pupils, as well as university students. experience normal menstruation and have no history of any psychiatric illness. HC participants were excluded (before scanning) if they; had any history of psychiatric illness, experience abnormal menstruation, had a lifetime BMI below the 10th age percentile (if younger than 18 years)/BMI below 18.5kg/m2 (if older than 18 years), or were currently obese (BMI over 97th age percentile if younger than 18 years; BMI over 30kg/m² if older than 18 years).

Further exclusion criteria for all participants were intelligence quotient (IQ) <85, psychotropic medication intake within four weeks prior to the study apart from certain antidepressants (patients were taking a selective serotonin reuptake inhibitor or a noradrenergic and specific serotonergic antidepressant), current substance abuse, inflammatory, neurologic or metabolic illness, chronic medical or neurological illness that could affect appetite, eating behaviour, or body weight, clinically relevant anaemia, pregnancy or breast feeding. Scans were also excluded from the analysis in case of significant neuroimaging artefacts/errors and if age > 23years. Anthropometric data included height and weight measurement directly before scanning, from which we calculated BMI and BMI-SDS.


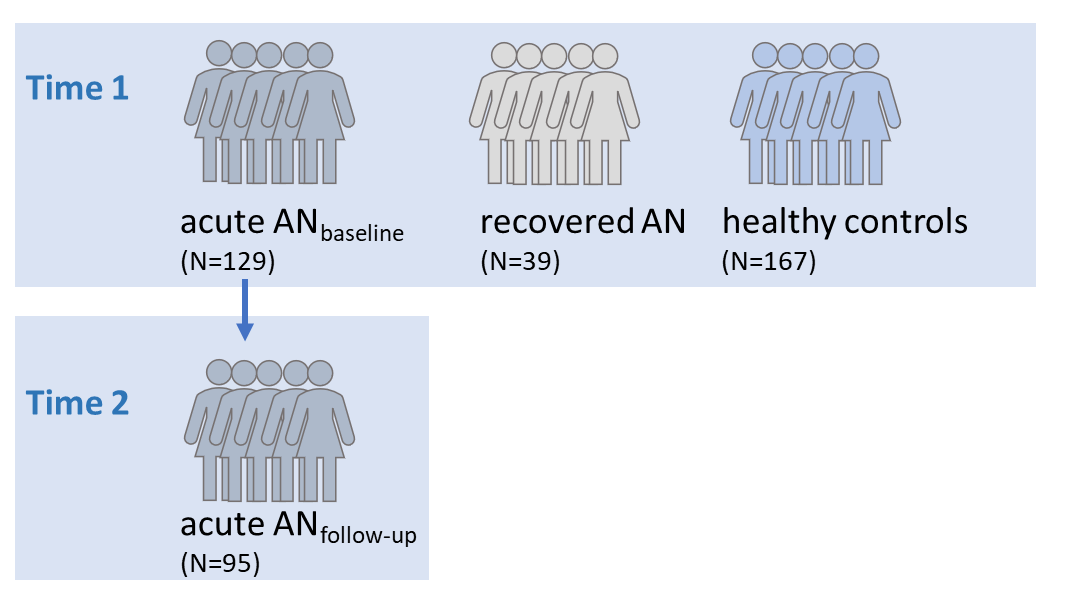


SM Figure 1. Study design.

***SM Section 1.2 Clinical Measures***

Exclusion criteria and possible confounding variables, e.g. the use of psychotropic medications and medical comorbidities, were obtained using the expert version of the Structured interview for anorexia and bulimia nervosa for DSM-IV [SIAB-EX^1^] and our own semi-structured interview. SIAB-EX is a well-validated 87-item semi-standardized interview that assesses the prevalence and severity of specific eating-related psychopathology over the past three months. The interview provides diagnoses according to the ICD-10 and DSM-IV and is suitable for adolescents as well as adults. It has been used widely in eating disorder research. A good inter-rater reliability (*k* =.81) for the diagnostic interview has been demonstrated.^1^ Interviews were conducted by clinically experienced and trained research assistants under the supervision of the attending child and adolescent psychiatrist.

For AN patients, comorbid psychiatric diagnoses other than eating disorders were derived from medical records and confirmed by an expert clinician with over 10 years of experience after careful chart review (including consideration of medical and psychiatric history, physical examination, routine blood tests, urine analysis, and a range of psychiatric screening instruments). All diagnostic information was ascertained at the time of treatment and the principal investigator of this study (SE) is also the chief consultant of the eating disorder treatment centre.

A German adaptation of the Eating Disorder Inventory-II (EDI-II^2,3^) was used to measure the severity of eating disorder symptoms and additional associated psychological traits. The EDI-II consists of 91 items and 11 positively correlated subscales including drive for thinness, body dissatisfaction, impulse regulation and perfectionism. Items are rated on a scale of 1 (never) to 6 (always). Items are summed to create a total score, with higher scores indicating greater severity of eating-disorder specific symptomology.

A German adaptation of the Beck Depression Inventory-II (BDI-II^4,5^) was used to measure depressive and affective symptoms. The BDI-II is a 21-item self-report measure that assesses major depressive symptoms. Each item is scored on a scale between 0 (never) to 3 (always). Items are summed to create a total score, with higher scores indicating high levels of depression. Illness duration was measured in years from initial onset. Demographic and clinical data were managed using a secure, web-based tool (Research Electronic Data Capture; <http://www.project-redcap.org>^6^).

***SM Section 1.3 Magnetic Resonance Imaging Acquisition and Processing***

All participants underwent MRI scanning between 8 and 9 a.m. following an overnight fast. High resolution 3D T1-weighted structural scans were acquired on a 3.0T scanner (Magnetom Trio, Siemens, Erlangen, Germany) using a rapid acquisition gradient echo (MP-RAGE) sequence with the following parameters: 176 sagittal slices (1 mm thickness, no gap), repetition time = 1900 ms; echo time = 2.26 ms; flip angle = 9° voxel size = 1.0 x 1.0 x 1.0 mm^3^; field of view = 256 x 224 mm^2^, bandwidth of 200 Hz/pixel.

**Supplemental Results**

***SM Section 2.1***

*Chronological age, BrainAGE_GM_ and BrainAGE_WM_ descriptives and correlations per group*


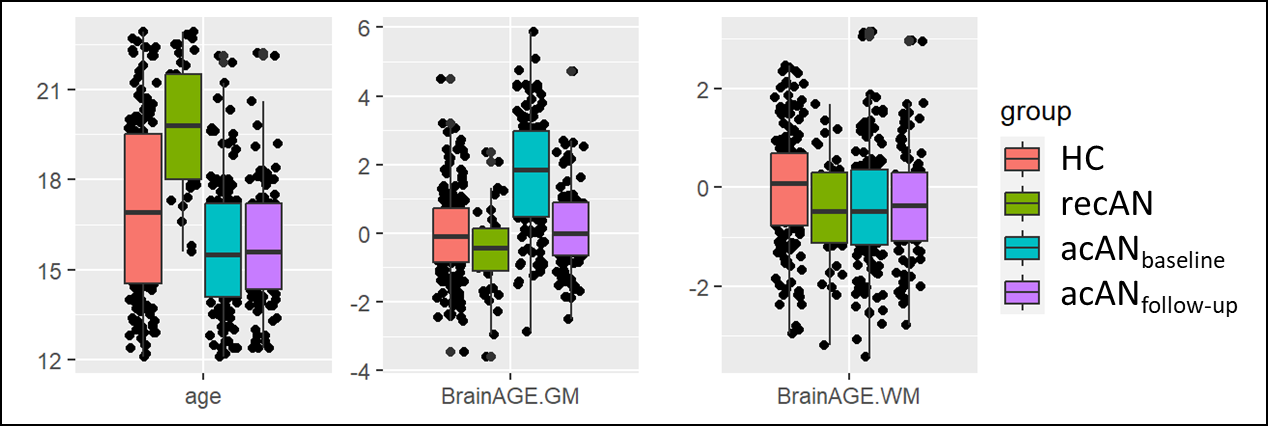


SM Figure 2. Chronological age, BrainAGE_GM_ and BrainAGE_WM_ per group. acAN_baseline_ = acute Anorexia Nervosa patients at admission; acAN_follow-up_ = acute Anorexia Nervosa patients after partial weight-recovery; recAN = recovered patients; HC = healthy controls; BrainAGE_GM_ = grey-matter brain-predicted age difference; BrainAGE_WM_ = white-matter brain-predicted age difference.


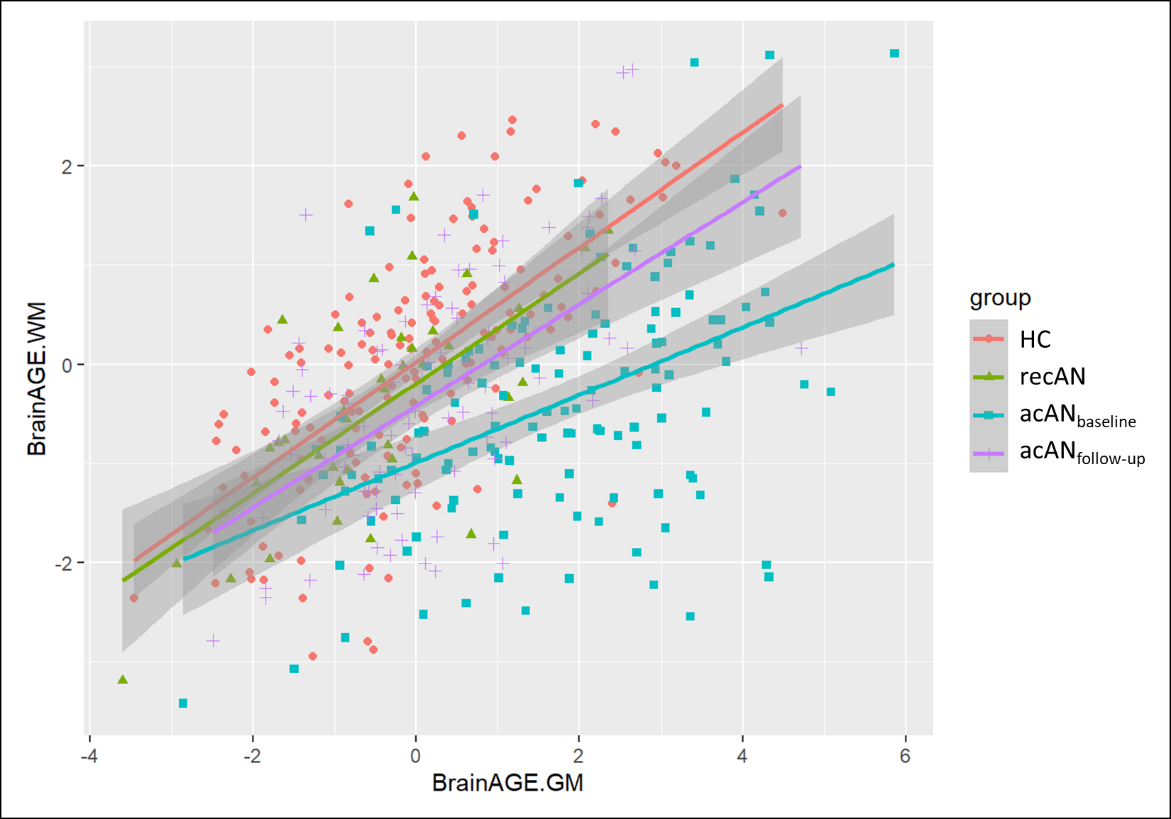


SM Figure 3. Correlations between grey- and white-matter-based BrainAGE per group. HC =healthy controls; acAN = acute AN patients; recAN = recovered AN patients.

***SM Section 2.2***

*Cross-sectional model 1, group differences in BrainAGE_GM_:*

|  | **BrainAGE GM** | | |
| --- | --- | --- | --- |
| *Predictors* | *Estimates* | *CI* | *p* |
| (Intercept) | -0.05 | -0.28 – 0.17 | 0.638 |
| group [recAN] | -0.43 | -0.94 – 0.09 | 0.103 |
| group [acAN_baseline_] | 1.79 | 1.45 – 2.13 | **<0.001*** |
| Observations | 335 | | |
| R^2^ / R^2^ adjusted | 0.283 / 0.279 | | |

SM Table S1. Group differences in BrainAGE_GM_. acAN = acute AN patients; recAN = recovered AN patients.

*Bold: nominally significant; * significant after multiple correction.*

*Cross-sectional model 2a, group differences in BrainAGE_GM_:*

|  | **BrainAGE GM** | | |
| --- | --- | --- | --- |
| *Predictors* | *Estimates* | *CI* | *p* |
| (Intercept) | 1.75 | 0.46 – 3.05 | **0.008*** |
| group [recAN] | 0.66 | -3.86 – 5.18 | 0.774 |
| group [acAN _baseline_] | 4.02 | 1.82 – 6.22 | **<0.001*** |
| age | -0.11 | -0.18 – -0.03 | **0.006*** |
| group [recAN] * age | -0.04 | -0.27 – 0.19 | 0.731 |
| group [acAN_baseline_] * age | -0.15 | -0.28 – -0.02 | **0.029** |
| Observations | 335 | | |
| R^2^ / R^2^ adjusted | 0.342 / 0.332 | | |

SM Table S2. Group differences in BrainAGE_GM_ remained stable when additionally covarying for age and age by group interaction. acAN = acute AN patients; recAN = recovered AN patients.

*Bold: nominally significant; * significant after multiple correction.*

*Cross-sectional model 2b, group differences in BrainAGE_GM_:*

|  | **Brain AGE GM** | | |
| --- | --- | --- | --- |
| *Predictors* | *Estimates* | *CI* | *p* |
| (Intercept) | 1.86 | 0.45 – 3.26 | **0.010** |
| group [recAN] | 0.88 | -4.06 – 5.81 | 0.727 |
| group [acAN _baseline_] | 3.96 | 1.59 – 6.34 | **0.001*** |
| age | -0.11 | -0.19 – -0.03 | **0.007*** |
| type of cur rec medication SSRI [yes] | 0.84 | -0.85 – 2.53 | 0.329 |
| group [recAN] * age | -0.05 | -0.30 – 0.20 | 0.688 |
| group [acAN _baseline_] * age | -0.14 | -0.29 – 0.00 | 0.052 |
| Observations | 291 | | |
| R^2^ / R^2^ adjusted | 0.349 / 0.335 | | |

SM Table S3. Group differences in BrainAGE_GM_ remained stable when additionally covarying for age, age by group interaction and current or recent SSRI use. acAN = acute AN patients; recAN = recovered AN patients.

*Bold: nominally significant; * significant after multiple correction.*

*Cross-sectional model 2c, group differences in BrainAGE_GM_:*

|  | **Brain AGE GM** | | |
| --- | --- | --- | --- |
| *Predictors* | *Estimates* | *CI* | *p* |
| (Intercept) | 2.12 | 0.37 – 3.86 | **0.018** |
| group [recAN] | 1.11 | -3.84 – 6.06 | 0.659 |
| group [acAN _baseline_] | 3.94 | 1.52 – 6.36 | **0.002** |
| age | -0.11 | -0.19 – -0.03 | **0.008** |
| siabex result an type [restrictive subtype] | -0.28 | -1.30 – 0.74 | 0.589 |
| group [recAN] * age | -0.06 | -0.31 – 0.19 | 0.629 |
| group [acAN _baseline_] * age | -0.14 | -0.29 – 0.01 | 0.060 |
| Observations | 288 | | |
| R^2^ / R^2^ adjusted | 0.346 / 0.332 | | |

SM Table S4. Group differences in BrainAGE_GM_ remained stable when additionally covarying for age, age by group interaction and AN subtype. acAN = acute AN patients; recAN = recovered AN patients.

*Bold: nominally significant; * significant after multiple correction.*

*Cross-sectional model 1, group differences in BrainAGE_WM_:*

|  | **BrainAGE WM** | | |
| --- | --- | --- | --- |
| *Predictors* | *Estimates* | *CI* | *p* |
| (Intercept) | -0.01 | -0.19 – 0.17 | 0.937 |
| group [recAN] | -0.45 | -0.87 – -0.03 | **0.034** |
| group [acAN_baseline_] | -0.39 | -0.67 – -0.12 | **0.005*** |
| Observations | 335 | | |
| R^2^ / R^2^ adjusted | 0.029 / 0.023 | | |

SM Table S5. Group differences in BrainAGE_WM_. acAN = acute AN patients; recAN = recovered AN patients.

*Bold: nominally significant; * significant after multiple correction.*

*Cross-sectional model 2a, group differences in BrainAGE_WM_:*

|  | **BrainAGE WM** | | |
| --- | --- | --- | --- |
| *Predictors* | *Estimates* | *CI* | *p* |
| (Intercept) | 1.52 | 0.44 – 2.60 | **0.006** |
| group [recAN] | -1.78 | -5.55 – 1.99 | 0.354 |
| group [acAN_baseline_] | -1.53 | -3.37 – 0.30 | 0.101 |
| age | -0.09 | -0.15 – -0.03 | **0.005*** |
| group [recAN] * age | 0.08 | -0.11 – 0.27 | 0.415 |
| group [acAN_baseline_] * age | 0.07 | -0.05 – 0.18 | 0.254 |
| Observations | 335 | | |
| R^2^ / R^2^ adjusted | 0.053 / 0.038 | | |

SM Table S6. Group differences in BrainAGE_WM_ when additionally covarying for age and age by group interaction. acAN = acute AN patients; recAN = recovered AN patients.

*Bold: nominally significant; * significant after multiple correction.*

*Cross-sectional model 2b, group differences in BrainAGE_WM_:*

|  | **Brain AGE WM** | | |
| --- | --- | --- | --- |
| *Predictors* | *Estimates* | *CI* | *p* |
| (Intercept) | 1.49 | 0.30 – 2.68 | **0.014** |
| group [recAN] | -1.13 | -5.30 – 3.05 | 0.596 |
| group [acAN_baseline_] | -1.71 | -3.72 – 0.30 | 0.095 |
| age | -0.09 | -0.16 – -0.02 | **0.011** |
| type of cur rec medication SSRI [yes] | -0.11 | -1.54 – 1.32 | 0.878 |
| group [recAN] * age | 0.05 | -0.16 – 0.26 | 0.645 |
| group [acAN_baseline_] * age | 0.08 | -0.04 – 0.20 | 0.202 |
| Observations | 291 | | |
| R^2^ / R^2^ adjusted | 0.044 / 0.023 | | |

SM Table S7. Group differences in BrainAGE_WM_ when additionally covarying for age, age by group interaction and current or recent SSRI use. acAN = acute AN patients; recAN = recovered AN patients.

*Bold: nominally significant; * significant after multiple correction.*

*Cross-sectional model 2c, group differences in BrainAGE_WM_:*

|  | **Brain AGE WM** | | |
| --- | --- | --- | --- |
| *Predictors* | *Estimates* | *CI* | *p* |
| (Intercept) | 2.24 | 0.78 – 3.71 | **0.003** |
| group [recAN] | -1.08 | -5.23 – 3.06 | 0.608 |
| group [acAN.TP1] | -1.39 | -3.42 – 0.64 | 0.178 |
| age | -0.09 | -0.15 – -0.02 | **0.015** |
| siabex result an type [restrictive subtype] | -0.82 | -1.67 – 0.03 | 0.058 |
| group [recAN] * age | 0.05 | -0.16 – 0.26 | 0.660 |
| group [acAN.TP1] * age | 0.06 | -0.07 – 0.18 | 0.383 |
| Observations | 288 | | |
| R^2^ / R^2^ adjusted | 0.054 / 0.033 | | |

SM Table S8. Group differences in BrainAGE_WM_ when additionally covarying for age, age by group interaction and AN subtype. acAN = acute AN patients; recAN = recovered AN patients.

*Bold: nominally significant; * significant after multiple correction.*

***SM Section 2.3***

Cross-sectional model 3, association of BrainAGE_GM_ with clinical measures:

|  | **BrainAGE GM** | | |
| --- | --- | --- | --- |
| *Predictors* | *Estimates* | *CI* | *p* |
| (Intercept) | 6.59 | 4.08 – 9.10 | **<0.001*** |
| BMI_sds_ | -0.34 | -0.63 – -0.06 | **0.017** |
| EDI_total_ | -0.01 | -0.02 – 0.00 | 0.164 |
| BDI_total_ | 0.02 | -0.02 – 0.07 | 0.349 |
| durill | -0.05 | -0.28 – 0.18 | 0.672 |
| age | -0.29 | -0.44 – -0.13 | **<0.001*** |
| Observations | 108 | | |
| R^2^ / R^2^ adjusted | 0.176 / 0.136 | | |

SM Table S9. Cross-sectional association of BrainAGE_GM_ with clinical measures. BMI_sds_ = standardized deviation score of body mass index; EDI_total_ = total score on the Eating Disorder Inventory; BDI_total_ = total score on the Beck Depression Inventory; durill = duration of illness.

*Bold: nominally significant; * significant after multiple correction.*

Cross-sectional model 3, association of BrainAGE_WM_ with clinical measures:

|  | **BrainAGE WM** | | |
| --- | --- | --- | --- |
| *Predictors* | *Estimates* | *CI* | *P* |
| (Intercept) | 0.04 | -1.97 – 2.06 | 0.965 |
| BMI_sds_ | 0.22 | -0.01 – 0.45 | 0.060 |
| EDI_total_ | -0.01 | -0.01 – 0.00 | 0.281 |
| BDI_total_ | 0.03 | -0.01 – 0.06 | 0.170 |
| durill | 0.02 | -0.16 – 0.20 | 0.841 |
| age | 0.05 | -0.08 – 0.17 | 0.451 |
| Observations | 108 | | |
| R^2^ / R^2^ adjusted | 0.056 / 0.009 | | |

SM Table S10. Cross-sectional association of BrainAGE_WM_ with clinical measures. BMI_sds_ = standardized deviation score of body mass index; EDI_total_ = total score on the Eating Disorder Inventory; BDI_total_ = total score on the Beck Depression Inventory; durill = duration of illness.

*Bold: nominally significant; * significant after multiple correction.*

***SM Section 2.4***

*Longitudinal change in BrainAGE_GM_, model 1:*

|  | **BrainAGE GM** | | |
| --- | --- | --- | --- |
| *Predictors* | *Estimates* | *CI* | *p* |
| (Intercept) | 1.85 | 1.56 – 2.14 | **<0.001*** |
| group [acAN_follow-up_] | -1.69 | -1.93 – -1.46 | **<0.001*** |
| **Random Effects** | | | |
| σ^2^ | 0.66 | | |
| τ_00_ _participant_id.ba_ | 1.40 | | |
| ICC | 0.68 | | |
| N _participant_id.ba_ | 95 | | |
| Observations | 190 | | |
| Marginal R^2^ / Conditional R^2^ | 0.259 / 0.762 | | |

SM Table S11. Longitudinal change in BrainAGE_GM._ Time point was modelled as a categorical variable (i.e. group = acAN_baseline_ and acAN_follow-up_). acAN = acute AN patients.

*Bold: nominally significant; * significant after multiple correction*.

*Longitudinal change in BrainAGE_GM_, model 2:*

|  | **BrainAGE GM** | | |
| --- | --- | --- | --- |
| *Predictors* | *Estimates* | *CI* | *p* |
| (Intercept) | 3.50 | 1.64 – 5.36 | **<0.001*** |
| group [acAN_follow-up_] | -1.72 | -1.97 – -1.47 | **<0.001*** |
| BMI.sds_baseline_ | -0.02 | -0.29 – 0.25 | 0.886 |
| BDI.total_baseline_ | 0.03 | -0.01 – 0.08 | 0.143 |
| EDI.total_baseline_ | -0.01 | -0.02 – -0.00 | **0.022** |
| durill_baseline_ | 0.14 | -0.12 – 0.41 | 0.299 |
| **Random Effects** | | | |
| σ^2^ | 0.70 | | |
| τ_00_ _participant_id.ba_ | 1.37 | | |
| ICC | 0.66 | | |
| N _participant_id.ba_ | 84 | | |
| Observations | 168 | | |
| Marginal R^2^ / Conditional R^2^ | 0.304 / 0.765 | | |

SM Table S12. Longitudinal change in BrainAGE_GM_ covarying for clinical measures at baseline. Time point was modelled as a categorical variable (i.e. group = acAN_baseline_ and acAN_follow-up_). acAN = acute AN patients. BMI_sds_ = standardized deviation score of body mass index; EDI_total_ = total score on the Eating Disorder Inventory; BDI_total_ = total score on the Beck Depression Inventory; durill = duration of illness.

*Bold: nominally significant; * significant after multiple correction.*

*Longitudinal change in BrainAGE_WM_, model 1:*

|  | **BrainAGE WM** | | |
| --- | --- | --- | --- |
| *Predictors* | *Estimates* | *CI* | *p* |
| (Intercept) | -0.44 | -0.68 – -0.21 | **<0.001*** |
| group [acAN_follow-up_] | 0.11 | 0.02 – 0.20 | **0.018** |
| **Random Effects** | | | |
| σ^2^ | 0.10 | | |
| τ_00_ _participant_id.ba_ | 1.23 | | |
| ICC | 0.93 | | |
| N _participant_id.ba_ | 95 | | |
| Observations | 190 | | |
| Marginal R^2^ / Conditional R^2^ | 0.002 / 0.926 | | |

SM Table S13. Longitudinal change in BrainAGE_WM_. Time point was modelled as a categorical variable (i.e. group = acAN_baseline_ and acAN_follow-up_). acAN = acute AN patients.

*Bold: nominally significant; * significant after multiple correction.*

*Longitudinal change in BrainAGE_WM_, model 2:*

|  | **BrainAGE WM** | | |
| --- | --- | --- | --- |
| *Predictors* | *Estimates* | *CI* | *p* |
| (Intercept) | 0.53 | -1.09 – 2.16 | 0.519 |
| group [acAN_follow-up_] | 0.11 | 0.03 – 0.19 | **0.006*** |
| BMI.sds_baseline_ | 0.21 | -0.03 – 0.44 | 0.088 |
| BDI.total_baseline_ | 0.02 | -0.02 – 0.06 | 0.291 |
| EDI.total_baseline_ | -0.00 | -0.01 – 0.00 | 0.314 |
| durill_baseline_ | 0.15 | -0.08 – 0.38 | 0.201 |
| **Random Effects** | | | |
| σ^2^ | 0.07 | | |
| τ_00_ _participant_id.ba_ | 1.28 | | |
| ICC | 0.95 | | |
| N _participant_id.ba_ | 84 | | |
| Observations | 168 | | |
| Marginal R^2^ / Conditional R^2^ | 0.072 / 0.952 | | |

SM Table S14. Longitudinal change in BrainAGE_WM_ covarying for clinical measures at baseline. Time point was modelled as a categorical variable (i.e. group = acAN_baseline_ and acAN_follow-up_). acAN = acute AN patients. BMI_sds_ = standardized deviation score of body mass index; EDI_total_ = total score on the Eating Disorder Inventory; BDI_total_ = total score on the Beck Depression Inventory; durill = duration of illness.

*Bold: nominally significant; * significant after multiple correction.*

***SM Section 2.4***

*Longitudinal change in BrainAGE_GM_, model 3:*

|  | **BrainAGE GM** | | |
| --- | --- | --- | --- |
| *Predictors* | *Estimates* | *CI* | *p* |
| (Intercept) | 0.80 | -0.45 – 2.06 | 0.210 |
| Change in BMI_sds_ | -0.65 | -0.75 – -0.54 | **<0.001*** |
| Change in BDI_total_ | 0.02 | -0.00 – 0.05 | 0.091 |
| Change in EDI_total_ | -0.01 | -0.01 – -0.00 | **0.048** |
| durill_baseline_ | -0.01 | -0.27 – 0.25 | 0.952 |
| **Random Effects** | | | |
| σ^2^ | 0.55 | | |
| τ_00_ _participant_id.ba_ | 1.63 | | |
| ICC | 0.75 | | |
| N _participant_id.ba_ | 91 | | |
| Observations | 168 | | |
| Marginal R^2^ / Conditional R^2^ | 0.318 / 0.829 | | |

SM Table S15. Longitudinal change in BrainAGE_GM_ predicted by change in clinical measures. BMI_sds_ = standardized deviation score of body mass index; EDI_total_ = total score on the Eating Disorder Inventory; BDI_total_ = total score on the Beck Depression Inventory; durill = duration of illness at baseline.

*Bold: nominally significant; * significant after multiple correction.*

*Longitudinal change in BrainAGE_WM_, model 3:*

|  | **BrainAGE WM** | | |
| --- | --- | --- | --- |
| *Predictors* | *Estimates* | *CI* | *p* |
| (Intercept) | -0.71 | -1.36 – -0.06 | **0.033** |
| Change in BMI_sds_ | 0.04 | -0.01 – 0.08 | 0.085 |
| Change in BDI_total_ | 0.00 | -0.01 – 0.01 | 0.775 |
| Change in EDI_total_ | 0.00 | -0.00 – 0.00 | 0.713 |
| durill_baseline_ | 0.13 | -0.07 – 0.34 | 0.208 |
| **Random Effects** | | | |
| σ^2^ | 0.06 | | |
| τ_00_ _participant_id.ba_ | 1.27 | | |
| ICC | 0.95 | | |
| N _participant_id.ba_ | 91 | | |
| Observations | 168 | | |
| Marginal R^2^ / Conditional R^2^ | 0.018 / 0.953 | | |

SM Table S16. Longitudinal change in BrainAGE_WM_ predicted by change in clinical measures. BMI_sds_ = standardized deviation score of body mass index; EDI_total_ = total score on the Eating Disorder Inventory; BDI_total_ = total score on the Beck Depression Inventory; durill = duration of illness at baseline.

*Bold: nominally significant; * significant after multiple correction.*

**Supplemental References**

1 Fichter M, Quadflieg N. The structured interview for anorexic and bulimic disorders for DSM-IV and ICD-10 (SIAB-EX): reliability and validity. *Eur Psychiatry* 2001; **16**: 38–48.

2 Paul T, Thiel A. *Eating Disorder Inventory-2 (EDI-2)*. Hogrefe, 2005.

3 Garner DM. *Eating Disorder Inventory-2*. Psychological Assessment Resources, 1991.

4 Hautzinger M, Kühner C, Feller F. *BDI-II Beck-Depressions-Inventar*. Pearson Assessment & Information GmbH, 2009.

5 Beck AT, Steer RA, Brown GK. *BDI-II, Beck depression inventory: manual*. (2nd ed.) Psychological Corp. ; Harcourt Brace, 1996.

6 Harris PA, Taylor R, Thielke R, Payne J, Gonzalez N, Conde JG. Research electronic data capture (REDCap)--a metadata-driven methodology and workflow process for providing translational research informatics support. *J Biomed Inform* 2009; **42**: 377–81.
